# Transient Neonatal Myasthenia Gravis as a Common Complication of a Rare Disease: A Systematic Review

**DOI:** 10.3390/jcm13041136

**Published:** 2024-02-17

**Authors:** Jenny Linnea Victoria Lindroos, Marte-Helene Bjørk, Nils Erik Gilhus

**Affiliations:** 1 Department of Clinical Medicine, University of Bergen, 5020 Bergen, Norway; jenny.lindroos@uib.no (J.L.V.L.); marte.bjork@uib.no (M.-H.B.); 2 Department of Neurology, Haukeland University Hospital, 5053 Bergen, Norway

**Keywords:** acetylcholine receptor antibodies, MuSK antibodies, myasthenia gravis, neonatal disease, maternal–fetal exchange, neuromuscular junction, autoantibodies

## Abstract

Myasthenia gravis (MG) is a rare autoimmune disease. Transient neonatal myasthenia gravis (TNMG) is caused by pathogenic maternal autoantibodies that cross the placenta and disrupt signaling at the neuromuscular junction. This is a systematic review of this transient immunoglobulin G (IgG)-mediated disease. TNMG affects 10–20% of children born to mothers with MG. The severity of symptoms ranges from minor feeding difficulties to life-threatening respiratory weakness. Minor symptoms might go unnoticed but can still interfere with breastfeeding. Acetylcholine-esterase inhibitors and antibody-clearing therapies such as immunoglobulins can be used to treat TNMG, but most children do well with observation only. TNMG is self-limiting within weeks as circulating antibodies are naturally cleared from the blood. In rare cases, TNMG is associated with permanent skeletal malformations or permanent myopathy. The mother’s antibodies can also lead to spontaneous abortions. All healthcare professionals meeting pregnant or birthing women with MG or their neonates should be aware of TNMG. TNMG is hard to predict. Reoccurrence is common among siblings. Pre-pregnancy thymectomy and intravenous immunoglobulins during pregnancy reduce the risk. Neonatal fragment crystallizable receptor (FcRn) blocking drugs for MG might reduce TNMG risk.

## 1. Introduction

Transient neonatal myasthenia gravis (TNMG) is an immunoglobulin G (IgG)-mediated disease caused by the maternal-to-fetal transmission of autoantibodies against functional proteins at the neuromuscular junction [1]. TNMG is the most common neonatal illness caused by the maternal-to-fetal disease transfer of myasthenia gravis (MG). This disorder is believed to affect 10–20% of children of mothers with MG [2]. Acetylcholine receptor (AChR) antibodies are the most common autoantibodies known to cause TNMG; however, muscle-specific kinase (MuSK) antibodies can cause this too.

TNMG is a treatable disease. It can vary in severity from mild to life-threatening [1]. Worries about passing on myasthenic weakness to the child are common in women with MG. In a study, 80% of women with MG who had not yet completed their family planning reported that they had abstained from having a child because of their MG [3].

The aim of this review is to discern the current knowledge and the knowledge gaps of TNMG and provide an overview of this disease’s concept in light of the present understanding of its mechanisms. We discuss clinical challenges and opportunities with a special emphasis on prediction and prevention, including a new promising treatment strategy for blocking neonatal fragment crystallizable receptors (FcRns).

## 2. Methods

**Figure A1:**
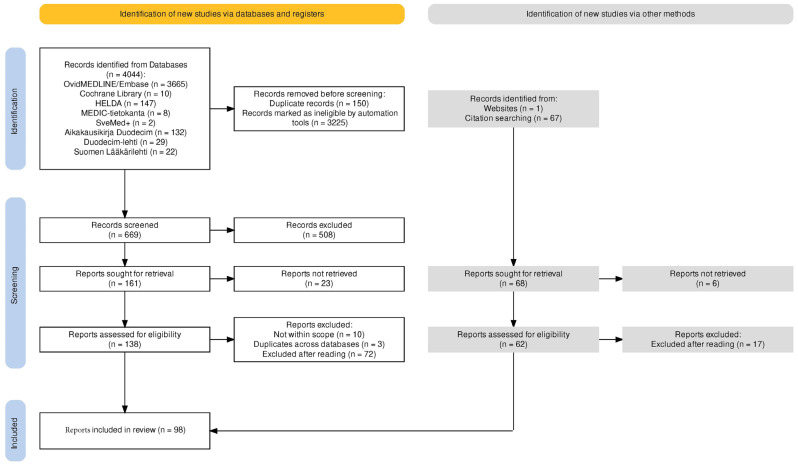
PRISMA flow diagram.

We conducted a systematic search by searching the Ovid Medline 1946 to September 2023, Embase 1974 to September 2023, and Cochrane Library databases using the keywords myasthenia gravis and neonate with synonyms and wildcard characters. A PRISMA flow diagram (Figure A1 [4]) and details (Table A1) are provided in accordance with PRISMA guidelines (Appendix A) [5]. We included all types of articles, including reviews, abstracts, and case reports. We reviewed the title and abstract of 300 papers, of which 152 papers were selected for full-text extraction. This compilation was carried out by the first author. We were confined to papers available through our institutions and were restricted by language (English, Finnish, or Scandinavian). We carried out additional searches in Scandinavian and Finnish databases, and we received auto-alerts on the above-mentioned keywords until 30 November 2023. Furthermore, we carried out a screening of reference lists and the online fields “similar articles” and “cited by”. We also included 61 papers from our previous acquaintance with MG in the literature.

## 3. Disease Concept

### 3.1. Classification

TNMG is defined in the 11th revision of the International Classification of Diseases (ICD-11; KB08.0) as “a pediatric condition characterized as a temporary autoimmune neuromuscular disease leading to fluctuating muscle weakness and fatigue in a newborn”.

TNMG should not be confused with congenital myasthenic syndromes (CMSs), which comprise a heterogenous group of rare hereditary conditions caused by genetic defects in functionally important molecules at the neuromuscular junction [6]. TNMG should also not be confused with juvenile MG, which is an acquired autoimmune disease like adult MG, but in the pediatric population [7]. In juvenile MG, there is a true de novo activation of the immune system, unlike the passively transferred autoimmunity in TNMG. Juvenile MG symptoms typically start after the age of 5 years and very rarely before the age of 2 years [8]. Neonatal onset, on the other hand, is typical for CMS [6].

TNMG and neonatal myasthenia gravis (NMG) are often used interchangeably. NMG has been recognized for more than 80 years [9,10]. TNMG is the “typical” expression of NMG. “Atypical NMG” has been used to describe a small subgroup of maternal MG-exposed children with additional persistent features and presumed antenatal NMG onset. This term has also been applied to children with TNMG-like symptoms lasting longer than the expected and typical 4-month mark.

Antenatal NMG onset can result in joint contractures as a consequence of fetal hypokinesia. Arthrogryposis multiplex congenita (AMC) is a rare congenital disorder characterized by multiple contractures. AMC is caused by decreased fetal movement, for which the etiology is heterogenous [2]. AMC has an incidence of 1:12,000 births [11] but is much more common with maternal MG. Severe skeletal anomalies were found in 2.2% of a nationwide MG birth cohort [12]. In the most severe form of AMC (known as fetal akinesia deformation sequence), lung maturation is affected due to fetal bulbar paresis with a lethal outcome [13]. Talipes equinovarus, which as an isolated anomaly is common and benign, can however, if identified in utero, be the first sign of developing AMC [14].

Fetal acetylcholine receptor inactivation syndrome (FARIS) is another expression of atypical NMG with antenatal onset. FARIS is distinguished by permanent myopathy, mainly of the facial and bulbar muscles. FARIS is linked to antibodies blocking the fetal type of AChR (fAChR), thus interfering with early fetal development [15,16]. FARIS often coexists with AMC, and thus, another term, fetal acetylcholine receptor antibody-related disorder (FARAD), has been suggested for these conditions [17]. FARAD children almost invariably show initial signs of TNMG, underscoring the shared disease mechanisms: exposure to maternal AChR antibodies. Additional anomalies, such as hearing loss, diaphragma paresis, CNS involvement, and pyloric stenosis, are associated with FARAD [17].

### 3.2. Epidemiology

MG is a rare disease affecting 1.2:10,000 females below the age of 50 years in the European population [18,19]. In a recent population-based cohort study in the United States, maternal MG was recognized in 1:10,000 deliveries [20]. TNMG affects 15–17% of infants born to mothers with MG based on recent systematic reviews [21,22]. An earlier TNMG rate based on a comprehensive review was 21% [10].

The proportion of maternal MG-exposed children diagnosed with TNMG ranged from 2.5% to 35% [23,24,25,26]. The disparity of reported TNMG rates probably reflects differences between studied populations and diagnostic accuracy. Many MG cohorts are compiled from highly specialized centers. This might give higher incidence estimates due to the selection of more severe MG patients. On the other hand, incidence estimates based on registry data can underestimate TNMG. In a nationwide cohort of MG pregnancies, only 4% of maternal MG-exposed children were captured with a TNMG diagnosis in the mandatory medical birth registry of Norway. However, 37% were transferred to a pediatric department, compared to only 2% of the reference group [24]. The TNMG estimate rose to 19% when using registry data combined with clinical records [27]. In a Swedish study using national health registries, TNMG was recorded in only 2.7% of MG births, but the authors suspected the true incidence to be around 8% by including infants registered with “congenital hypotonia, respiratory distress, and suspected nervous system disorder” [25]. Lower incidence rates in recent studies could be a reflection of more aggressive disease-modifying MG treatment and the earlier thymectomy of women of childbearing age.

### 3.3. Clinical Presentation and Evaluation

#### 3.3.1. Symptoms

The clinical picture of TNMG is dominated by hypotonic muscle weakness, which is most prominent in the bulbar muscles, face, and neck. Presenting symptoms are usually feeding difficulties, a weak cry, and sometimes problems with breathing [28]. The severity of respiratory insufficiency can range from mild to severe, from intermittent desaturations to life-threatening respiratory arrest. Intubation and mechanical ventilation are rarely needed [28].

Generalized hypotonia in the neonate can be noted by the “frog position” and poor head control. Facial weakness is characterized by ptosis, an open mouth, and hypomimia. Ptosis and ophthalmoparesis are less prominent in TNMG than in adult MG [29].

Feeding difficulties in TNMG are characterized by weak sucking and swallowing and fatigue during eating. This can result in a struggle at the breast, regurgitations, dehydration, and poor weight gain. Tube feeding is sometimes necessary during the first days or weeks. A high number (24%) of neonates born to mothers with MG had icterus in a recent single-center cohort [30], which might be a sign of inadequate hydration.

There is no widely used scale for grading TNMG severity. We suggest using a pragmatic grading of symptoms to guide treatment (Table 1). A list of grading systems used by other authors is available in Appendix A (Table A2).

Approximately one-half of TNMG cases presents at birth. Symptom onset is often delayed by 6 to 72 h [31]. Even with delayed onset, 80% present within 24 h postpartum [32]. In a few cases, the initial symptom-free period lasts up to 4 days [28,33].

#### 3.3.2. Prognosis

TNMG lasts only for as long as the transmitted antibodies persist in the child’s blood circulation. The mean duration of TNMG symptoms is 2–3 weeks. Complete resolution is observed in less than 2 months in 90% of children. The remaining 10% recover spontaneously within 4 months [32]. The empirically observed time course of TNMG matches the theoretically calculated time for IgG clearance, 15 weeks, which is equal to five times the normal half-life of IgG [34].

### 3.4. Long-Term Outcome and Persistent Myopathic Sequela

TNMG is defined as a transient neonatal disease. A lack of improvement after 1–2 months or any symptom residue beyond 4 months should prompt the clinician to consider alternative diagnoses or comorbidities. CMS is a relevant differential diagnosis if the mother does not have a definitely confirmed MG. The CMS of the fast channel type can partly respond to acetylcholine esterase inhibitors, and misdiagnosis is not uncommon [35].

Children exposed to antibodies with a high affinity for fAChR in utero can develop FARIS. FARIS is usually seen following TNMG, with or without AMC. There is persistent muscle weakness, mainly in the bulbar region. This myopathy can be disabling or only mild [36,37,38].

Myopathic sequela has been seen in a MuSK-antibody seropositive child with severe TNMG. The child had a mild delay in motor development and started walking unaided at 20 months but eventually met all motor milestones [39].

FARIS and myopathic sequela after TNMG may be underrecognized [17,37]. In a Swedish follow-up study from 1992, a cohort of 31 children of 15 mothers with MG during pregnancy were examined with clinical and neurophysiologic tests conducted at an average age of 10 years [40]. Eleven of the children had had TNMG in the newborn period, and two of these, a sibling pair, had persistent weakness in the facial muscles compatible with myopathy on electromyography. This illustrates the pathophysiological overlap between TNMG and FARIS/FARAD. We recommend that attention be given to possible permanent myopathic sequela in all children with maternal MG exposure, especially those with severe TNMG or with a sibling with AMC or FARIS/FARAD.

### 3.5. Pathophysiology

TNMG is directly tied to maternal MG. The maternofetal transmission is mainly placental [1]. The pathogenicity of autoantibodies in MG [35] and NMG has been clearly demonstrated [41]. However, antibody transmission is not the only determinant of TNMG. The antibody titer in the newborn correlates well with the maternal antibody titer, regardless of TNMG symptom expression [42]. In addition to maternal MG exposure, the outcome of the child is determined by both fetal and placental factors [43]. This is illustrated by case reports where only one twin had TNMG [44,45].

#### 3.5.1. Maternally Produced Autoantibodies

AChR antibodies are found in 80–90% of women of childbearing age with MG using routine assays. Early-onset MG with thymic hyperplasia is the most common MG subtype in this population [2]. Seronegative MG is believed to be caused by autoantibodies that could be uncovered using more sensitive methods [46]. Up to 50% of patients with purely ocular MG are seronegative, but many convert to generalized MG and develop measurable antibodies over time [47].

**Figure 1:**
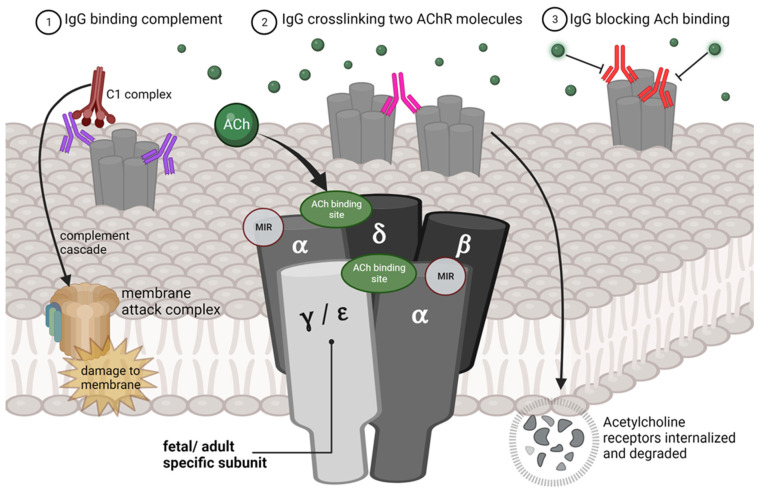
Pathogenic mechanisms of immunoglobulin G (IgG) antibodies (depicted in purple, pink and red) against the acetylcholine receptor (AChR), in relation to the fetal/adult AChR subunit structure, localization of the main immunogenic region (MIR), and the acetylcholine (Ach) binding site.

AChR antibodies can induce muscle weakness through at least three mechanisms (Figure 1): (1) damaging the postsynaptic membrane folds through complement activation, (2) promoting receptor degradation by crosslinking two AChR molecules, and (3) directly inhibiting neuromuscular transmission by blocking the acetylcholine (Ach)-binding site [35]. Complement-mediated destruction is probably the most important mechanism in generalized AChR-antibody-mediated MG but could be less important in TNMG, as the complement system is immature at birth [43]. On the other hand, the maternal complement system is overactivated in pregnancy, and this is believed to be a disease-inducing factor in some other disorders with maternal-to-fetal antibody transmission [48]. AChR antibodies with a direct blocking action are rare but have been associated with AMC [46] as well as TNMG [42]. Another TNMG child had both modulating- and blocking-type AChR antibodies [49]. Routine assays do not discriminate between different AChR antibody subtypes.

**Figure 2:**
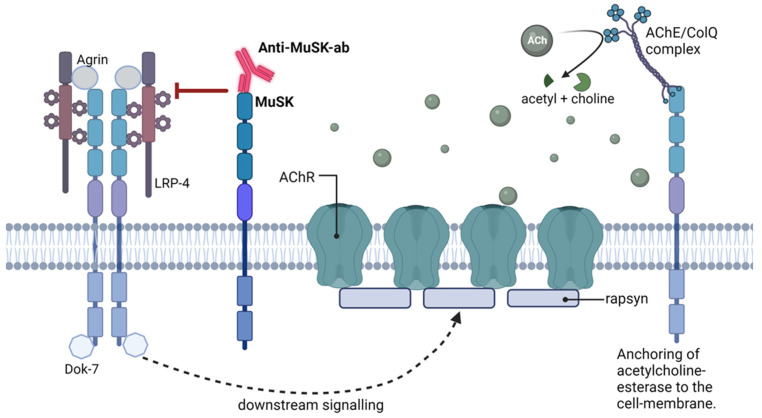
Antibodies against MuSK inhibit the formation of the MuSK-LRP4-Agrin complex and prevent the activation of intracellular pathways that normally lead to AChR clustering. MuSK is also responsible for anchoring acetylcholine-esterase to the postsynaptic membrane. Abbreviations: MuSK; muscle-specific kinase. LRP4; low-density lipoprotein receptor-related protein 4. AChR; acetylcholine receptor. Ach; acetylcholine. AChE; acetylcholine esterase. ColQ; collagen-like tail subunit of asymmetric acetylcholinesterase.

MuSK autoantibodies (Figure 2) are found in up to one-third of AChR seronegative MG patients. MuSK-MG has been associated with TNMG [50], illustrating that MuSK antibodies have a pathogenic ability similar to AChR antibodies. MuSK-TNMG, like adult MUSK-MG, is often a severe disease with prominent bulbar affection and respiratory insufficiency [51].

TNMG has also been described in the children of mothers with purely ocular MG or double-seronegative MG (i.e., AChR-seronegative and MuSK-seronegative MG) [33,52]. Low-density lipoprotein receptor-related protein 4 (LRP4) antibodies have been seen in some double-seronegative MG patients, but their pathogenicity is not certain. To our notion, no LRP4-associated TNMG case has yet been recorded, but testing for LRP4 antibodies is not widespread.

A TNMG-like disorder has been described in a few cases where the mother had Lambert–Eaton myasthenic syndrome. This disorder is caused by IgG antibodies against presynaptic voltage-gated calcium channels (VGCCs) at the neuromuscular junction [53].

#### 3.5.2. Maternal-to-Fetal IgG Transmission

The antibody repertoire of the neonate usually mirrors that of the mother. This holds true both for TNMG-affected children, asymptomatic children with maternal MG exposure [54], and healthy mother–children pairs [55]. It is not known why only a minority of children exposed to AChR and MuSK antibodies in utero develop symptoms.

**Figure 3:**
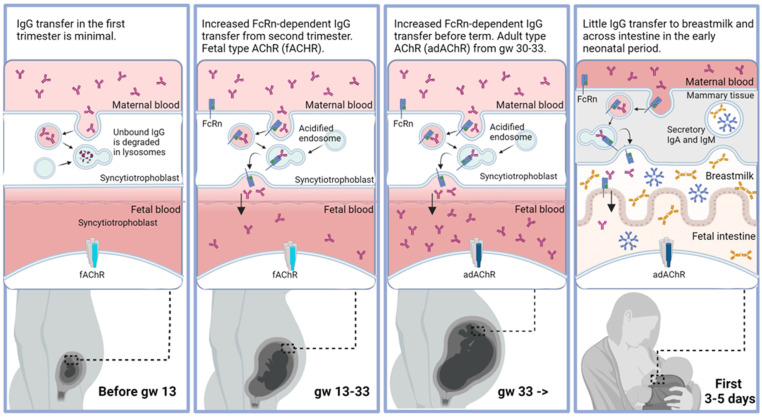
Neonatal fragment crystallizable receptor (FcRn)-dependent transmission of pathological IgG from mother to child and AChR antibody targets (fAChR depicted in light blue-grey and adAChR depicted in dark blue-grey) according to the fetal/neonatal developmental stage, illustrating the mechanisms for the disease transfer of maternal MG in TNMG. Abbreviations: gw; gestational week.

The transplacental transfer of IgG is nearly non-existent until 10–12 weeks of gestation in normal pregnancies (Figure 3) [56]. During the second and third trimesters, IgG levels are continuously increasing in cord blood and reach a level of more than 50% of the maternal concentration by week 33. After gestational week 36 and towards term, there is a sharp increase in the total fetal IgG related to increased transplacental IgG1 transfer [57].

The transplacental transport of IgG is mediated by FcRn [56]. FcRn favors IgG1 and IgG4, and transport is less efficient for IgG3 and IgG2 [34]. AChR antibodies mainly belong to the IgG1 subclass and sometimes to IgG3 and IgG4 [48]. MUSK antibodies are usually of the IgG4 subclass [47]. FcRn selectively binds to IgG, not IgM, IgA, or IgE. This also makes IgG overrepresented in the blood circulation [34].

The FcRn-mediated transplacental transport represents a passive, pH-dependent mechanism [34]. Maternal IgG is first internalized in endothelial cells (syncytiotrophoblasts) lining the blood vessels on the maternal side of the placenta. The IgG-containing vesicles then fuse with endosomes, causing FcRn to bind to IgG due to a low pH environment. This binding prevents the degradation of IgG by lysosomes and results in the transcytosis of the endocytic vesicles to the fetal side of the placenta. As the vesicles fuse with the cellular membrane on the fetal side, FcRn automatically frees IgG due to the neutral pH in the extracellular space. IgG then passes across the placental interstitium and the endothelial cells lining the fetal blood vessels, probably through a similar FcRn-dependent mechanism.

The total IgG level in infant cord blood typically exceeds that of maternal levels by 20–30% at term [57]. In one study, the mean AChR antibody level of the newborns was 120% of the mothers’ [58]. In another study of 21 mothers and 26 infants, neonatal AChR-antibody titers at birth were slightly lower than maternal titers, except for one case where the child had more than twice the level of the mother [54]. Specific antibodies have different relative transfer rates. Low maternal IgG titers are compensated for by a higher maternal-to-fetal transfer rate. The specific antibody titer in the fetus can, therefore, exceed the maternal titer by more than 300% [57]. On the other hand, placental FcRn receptors can be saturated, and this results in lower transfer rates if maternal blood holds a high overall content of IgG [57]. This illustrates why the neonatal AChR antibody titer sometimes differs from the maternal. It also explains why the treatment of the mother with IVIG reduces the transplacental transfer of pathogenic autoantibodies [57].

#### 3.5.3. Fetal- and Adult-Type Acetylcholine Receptors

Muscle-type nicotinic AChR is a ligand-gated ion channel present in striated muscles and responsible for delivering the signal for muscle contraction. It appears in the following two forms: the fetal type (fAChR) and the adult type (adAChR). The fAChR is present in uninnervated muscles during fetal development. It is gradually replaced by the adAChR during gestational weeks 30–33 (Figure 3) [59].

The fAChR (α₂βγδ) and the adAChR(α₂βεδ) only differ in one of the five transmembrane subunits (Figure 1). The fetal form contains a γ-subunit instead of an adult ε-subunit. The two α-subunits, one β- and one δ-subunits are shared [59]. The fAChR has a shorter half-life (24 h) compared to adAChR (10 days) [35].

Most AChR antibodies bind to the main immunogenic region (MIR), which is localized on the two shared α-subunits, but AChR antibodies can target any of the five subunits [46]. An AChR antibody can also be cross-reactive, i.e., have affinity for several different epitopes [59]. Antibodies associated with TNMG typically show binding both to the fAChR and the adAChR [59]. Most AChR antibodies bind to both fAChR and adAChR through an affinity to shared epitopes [46]. AChR autoantibody populations are heterogenous, i.e., an AChR seropositive individual has an array of antibodies comprising antibody subpopulations with different AChR epitope specificities [60].

A small proportion of the autoantibodies are selective to the fetal γ-subunit or rarely to the adult ε-subunit. Antibodies with a high specificity for either the γ-subunit or the ε-subunit are more common in purely ocular MG [59]. Antibodies blocking the fAChR due to high affinity for the γ-subunit are linked to the antenatal damage seen in FARIS/FARAD [17]. Acetylcholine has two binding sites on the AChR: one at the αδ-site and one at the αγ/αε-site. Inhibitory antibodies binding to the αγ-site, thus, specifically block acetylcholine from binding to the fAChR and paralyze the child at an early stage of fetal development [17]. Inhibiting antibodies targeting the αε-site causes movement restriction only in the third trimester [59].

### 3.6. Prediction of TNMG

A previously affected sibling is a strong predictive factor for TNMG [1]. However, having a healthy child following a previous child with TNMG is still possible [61]. In a study of 31 children in 15 mothers, except for one family, either all or none of the siblings were affected [40].

There are no useful genetic markers for TNMG. HLA-typing proved not to be a predictive marker for TNMG [1,40].

The deterioration of the mother’s MG during pregnancy is associated with TNMG [62]. For stable MG, however, maternal disease severity [13] or duration are not reliable predictive factors for TNMG [23]. Asymptomatic mothers [13,63] and mothers in complete remission [64] can have children with TNMG. One woman who previously gave birth to a child with TNMG was in complete remission during her second pregnancy but still had a child with fetal akinesia deformation sequence (i.e., lethal AMC) [12]. Vice versa, women with advanced MG can have healthy children [13,42]. More severe maternal MG is associated with the more aggressive treatment of the mother. This could counteract the harm of maternal MG by reducing the antibody load on the child. This complex interplay between maternal disease activity and medication during pregnancy may obscure the relationship between maternal clinical status and TNMG occurrence.

The maternal absolute AChR antibody titer does not reliably predict TNMG. Although some studies have shown a weak correlation between the AChR antibody titer and TNMG [42,65], others have failed to establish this [13]. Mothers with very low AChR antibody titers can have severely affected children [66], and conversely, children can be healthy despite high maternal titers [42]. Very high titers may be indicative of TNMG, but there is no clear cut-off value. In one study with 21 MG mothers, 9/26 children had TNMG, and all but one of their mothers had AChR antibody titers above 37 nM, while all but one of the healthy children’s mothers had AChR antibody titers below 41 nM [54]. The antibody titers tend to decrease during pregnancy, but there is large intra- and inter-individual variation [67]. AChR and MuSK antibody titers are also of limited value in the clinical follow-up of adult MG patients [68]. AChR antibody titers show high variability between individuals with MG, although they tend to be lower in purely ocular MG. It could be that some specific antibody subtypes can cause TNMG at low concentrations, while high concentrations are necessary for other antibody subtypes. AChR-idiotypic antibodies can also occur together with anti-idiotypic antibodies, which may counteract the original autoantibodies [69]. The protective role of anti-idiotypic antibodies has been shown in maternal-neonatal lupus dyads [57]. Hence, the routine clinical testing of the total AChR antibody titer in the mother cannot be used to predict TNMG.

A high ratio of the fetal versus adult AChR antibody titer (fAChR/adAChR-ab ratio) was reported as indicative of TNMG [67,70]. Paradoxically, the fAChR/adAChR-ab ratio was equally low for asymptomatic children and for the most severely affected children with “antenatal onset NMG” (FARAD). However, excluding children with “atypical NMG” (FARAD), TNMG occurred only with a maternal fAChR/adAChR-ab ratio above 2.5 [70]. Low concentrations of certain antibody subtypes seem to be sufficient for the development of FARAD (antibodies blocking the fAChR). Since adAChR is predominant in the child after 33 weeks of gestation, one would expect that antibodies directed against this form are crucial for TNMG development. However, the association between a high fAChR/adAChR-ab ratio and TNMG contradicts this [67]. The antibodies targeting the fAChR may be essential in “priming” TNMG by interfering with the maturation of the neuromuscular junction.

Reduced fetal movement in utero suggests TNMG and is a risk factor for AMC. The reduced swallowing of amniotic fluid can result in polyhydramnios. Women with MG should be monitored with standard antenatal care screenings, including regular assessments of fetal growth, maternal self-monitoring of fetal movements from 24 weeks of gestation, and an ultrasound scan before 24 weeks of gestation as a minimum [71,72]. A rapid increase in the mother’s abdomen size or suspicion of reduced fetal movement should invoke additional follow-up. The maternal self-monitoring of fetal movement, with or without a daily movement counting method, is not a very sensitive method [72]. Only 14/36 mothers of children with AMC diagnosed in the fetal period had noted a decrease or absence of fetal movements [14]. Some recommend monthly ultrasound scans for women with MG [73,74]. The sensitivity for detecting AMC on a routine ultrasound is 50% but can be increased to over 80% with serial scans [14]. We strongly recommend serial prenatal ultrasound scans to assess fetal growth, amniotic fluid volume, fetal joint position, and motor function if the woman has had a previous child with TNMG, AMC, or previous spontaneous abortions, and it should be considered for all MG pregnancies.

### 3.7. Diagnostic Procedures

A typical clinical presentation, together with a history of maternal MG, is sufficient for diagnosing TNMG, regardless of the mother’s MG type or disease activity.

If the mother has not been diagnosed with MG, it is harder to establish the diagnosis. Other causes of neonatal hypotonia, feeding difficulties, and respiratory distress are far more common than TNMG. Furthermore, several life-threatening differential diagnoses must be excluded, such as perinatal asphyxia, hypovolemia, hypoglycemia, neonatal infections and sepsis, congenital heart disease, respiratory distress syndrome, and metabolic conditions.

The clinical neurological examination of a newborn with TNMG should disclose hypotonic muscle weakness. The weakness or fatiguability of the sucking reflex is a sensitive sign. Concomitant AMC with contractures needs to be discriminated from spastic paresis. The child should have normal alertness and responsiveness. Deep tendon reflexes should be intact. Spontaneous movements and newborn reflexes such as the Moro and grasp reflex can be weak due to floppiness.

A pharmacologic challenge test is a fast and easy way to confirm the diagnosis but can be falsely negative. A single dose of neostigmine (0.15 mg/kg administered intramuscularly (IM) or subcutaneously (SC)) is expected to produce a definite improvement of myasthenic muscle weakness within 10–15 min, which, for example, is observed by a stronger sucking reflex. The effect lasts up to 3 h [29]. The very short-acting acetylcholine-esterase inhibitor edrophonium is no longer recommended in newborns as respiratory arrest has been described [75].

Repetitive nerve stimulation (RNS) can be used for diagnosing TNMG but is cumbersome and rarely needed. The test requires an experienced neurophysiologist, is vulnerable to interpretation, and is invasive [71]. RNS is more sensitive and specific than the pharmacologic challenge test and can be useful in premature infants or those with neonatal comorbidities [76].

Serological testing for muscle antibodies in the newborn adds no clinical value if the mother has confirmed MG [71]. On the other hand, testing for AChR antibodies should always be performed in both mother and child in the absence of a maternal MG history. Detecting antibodies in the neonate can be hard as the titer drops rapidly after birth, but antibodies in the mother are sufficient for the diagnosis of TNMG. The prevalence of AChR antibodies is extremely low in the general population. We also recommend testing for MuSK antibodies if the clinical suspicion of TNMG is high. A correct diagnosis of TNMG is important even if the mother is asymptomatic and the child has recovered, as this has consequences for future pregnancies.

## 4. Management

Treatment should be guided by symptom severity (Table 1). The majority of TNMG patients present with mild disease requiring only attention to feeding, and the baby can be discharged home after 3 days of observation [29]. Treatment options are supportive/symptomatic or disease-modifying. There are no randomized controlled trials carried out in TNMG. With disease-modifying therapy, the intention is to accelerate the clearance of pathogenic antibodies. Immunosuppressants have no role in TNMG as the autoimmunity is passively transmitted, not inherent. The natural course of the disease, in combination with the maturation of the child, means that frequent reassessments of treatment necessity are required.

### 4.1. Supportive Treatment

Supportive treatment is the cornerstone regardless of TNMG severity. It comprises frequent small oral feedings or a nasogastric tube if necessary. Hydration is important. Respiratory support (continuous airway pressure, nasal oxygen supplement, or mechanical ventilation) is sometimes needed.

### 4.2. Pharmacological Treatment

Symptomatic treatment with acetylcholine-esterase inhibitors can be used for all grades of TNMG. Neostigmine and pyridostigmine are usually well tolerated and are administered as needed every 3–4 h parenterally (preferably IM or SC) or every 4–6 h enterally (per os (PO) or by nasogastric tube (NG)). Neostigmine (neostigmine methylsulfate: 0.05 mg/kg IV/IM/SC, neostigmine bromide: 0.5 mg/kg PO/NG) is usually preferred for parenteral use, while the slower-acting pyridostigmine is preferred for enteral use (0.05–0.15 mg/kg IV/IM or 0.5–1.0 mg/kg PO/NG, max. 10 mg/dose) [29,71,77]. Gradual upward titration and gradual tapering are recommended. It is sensible to schedule the administration prior to feedings for maximal benefit. The effect appears 15–30 min after enteral administration. Muscarinic side effects (diarrhea, increased secretions, fasciculations, and flushing) are dose-dependent and usually manageable by altering the dosing regimen. Increased bronchial secretions are a side-effect that limits their use in intubated patients. Anticholinergic agents like glycopyrronium bromide and atropine reverse the action of acetylcholine-esterase inhibitors.

### 4.3. Immunoglobulins and Plasmapheresis

IVIG and TPE should be considered in moderately and severely affected neonates. The clinical effect usually appears after 2–4 days [78,79]. The treatment can be repeated. Antibody titers can, in addition to clinical status, be used to monitor improvement. TPE should be reserved for the most severe cases [71]. MuSK-TNMG responds less efficiently to acetylcholine-esterase inhibitors [32,39]. Thus, the requirement for IVIG/TPE is often more pronounced in MuSK-TNMG.

### 4.4. Observation of Asymptomatic Newborns

All newborns to mothers with known MG should have access to prompt specialized neonatal evaluation and neonatal intensive care if needed. Conventional newborn care with early skin-to-skin care should be practiced when possible. All caregivers should be aware of the signs of TNMG, and the newborn should be observed in the hospital for at least 48 h [71], and ideally 72 h [19,28,33]. Some even recommend observation for 1 week [28,80]. We recommend that all asymptomatic children are observed in the hospital for at least 3 days, but 4 days if a previous sibling had TNMG or if pyridostigmine was given to the mother during delivery.

### 4.5. Special Considerations

Patients with impaired neuromuscular transmission, including newborns with TNMG [81], may experience the worsening of myasthenic symptoms due to certain drugs, such as aminoglycosides, ciprofloxacin, and anesthetic agents [82]. Non-depolarizing neuromuscular blocking agents can cause prolonged paralysis even after a single dose and interfere with recovery from anesthesia. Sugammadex has successfully been used to reverse this effect [49].

### 4.6. Breastfeeding and TNMG

TNMG can be an obstacle to breastfeeding due to weak sucking, lip weakness in keeping hold of the nipple, problems with positioning at the breast due to poor head control, and impaired swallowing. The breastfeeding success rate in MG was 76% in one study [26].

Breastfeeding should be encouraged in women with MG according to consensus guidelines [71]. Maternal MG pharmacotherapy is compatible with breastfeeding, with a few important exceptions: methotrexate, mycophenolate mofetil, and cyclophosphamide [2,71]. The increased risk for MG onset in the postpartum period might be ameliorated by breastfeeding [83].

The fact that TNMG often manifests after an initial symptom-free period has led to the concern that there is a transfer of pathogenic antibodies to the neonate through breastmilk. Some authors have, therefore, discouraged breastfeeding for women with MG [80,84], and some have suggested that breastfeeding should be avoided in newborns with TNMG [85]. The general benefits of breastfeeding are universally accepted. Breastmilk provides the newborn child with protective IgA and IgM, and breastfeeding strengthens the emotional bond between mother and child. Keeping in mind the transient and mostly benign nature of TNMG, we strongly recommend that women with MG are encouraged to breastfeed, but a careful approach is sensible for the first 3–4 days if the child has moderate or severe TNMG or if a previous sibling had severe TNMG.

Very low amounts of IgG are taken up in the gut by the newborn, and the absorption is believed to be significant only 1–2 days into the postnatal period [57,86,87]. A study found a 13% increase in neonatal serum-IgG in breastfed infants from birth to the 5th day of life, compared to a reduction of 24% in formula-fed infants (*p* < 0.001) [88]. Gastrointestinal IgG uptake in the child is likely FcRn-mediated, as FcRn is expressed in the fetal intestine [89]. The child swallows huge amounts of amniotic fluid, which contains IgG [87], and this can be an additional pathway for IgG transfer in utero, especially during early pregnancy, before the placenta is fully matured. However, most of the human maternofetal IgG transfer is placental as opposed to lactational, in contrast to other mammals [57]. Human breastmilk has a low concentration of IgG, which is only 4–6% of the total immunoglobulin count [87].

The IgG levels in breastmilk are highest just after birth and diminish quickly as colostrum is replaced by mature milk around day 3–4. IgG in breastmilk is partly secretory IgG that is produced locally in breast tissue and partly IgG from the systemic circulation [90]. FcRn is responsible for IgG transfer to breastmilk. Colostrum has an IgG concentration similar to the mother’s serum [88]. IgG1 levels range from 37 to 2800 mg/L in colostrum and 10–37 mg/L in the mature milk of healthy women [90]. The AChR antibody titer in the colostrum of two lactating MG women at 12 h postpartum was 14% and 20% of the mother’s serum concentration, respectively. The AChR antibody concentration showed, as expected, a rapid drop from day 1 to day 4 postpartum [91]. Thus, if there are no signs of TNMG after the in-hospital monitoring period, breastfeeding is safe. Breastfeeding is also recommended for children with TNMG after colostrum has been replaced with mature milk.

### 4.7. Prevention

Reducing the amount of circulating IgG autoantibodies in the mother reduces the antibody load in the child. This can be achieved with effective maternal immunosuppression, pre-pregnancy thymectomy, or antibody-clearing therapies (IVIG, TPE, or FcRn-blockers) during pregnancy. There is increasing evidence that this reduces the risk for TNMG, AMC, and FARIS/FARAD [13,17,26,36,66,92]. Low-dose corticosteroids and azathioprine are regarded as safe pharmacologic choices during pregnancy [19]. Methotrexate, mycophenolate mofetil, and cyclophosphamide are teratogenic and should be avoided [71]. 

Pre-pregnancy thymectomy reduces the risk for TNMG [19,26,93]. In a nationwide MG birth cohort, the TNMG prevalence was 13% if the mother was thymectomized compared to 27% if she was not (*p* = 0.03) [94]. Thymectomy is also associated with a better maternal MG status during pregnancy [95]. The effect of thymectomy appears gradually over several months. The procedure is not indicated during pregnancy, as this adds additional risks to the mother and the child. Thymectomy is recommended well before pregnancy in all AChR seropositive MG females with childbearing potential [19].

FcRn blockers comprise a promising new group of drugs approved for use in generalized MG. They have the capacity to reduce the amount of circulating IgG antibodies. In addition, they effectively restrict the passage of IgG across the placenta [56]. Thereby, they have the potential for use during pregnancy to prevent the maternal-to-fetal transmission of IgG antibody-mediated diseases like TNMG [19,56]. They have been shown to efficiently block maternal-to-fetal IgG transfer in vitro [96] and in vivo in an AMC mouse model [97]. The treatment was also effective at reducing AMC deformities. Animal experiments did not show undesirable effects on pregnancy or fetal development [97].

Candidates for FcRn blockade could be women with MG and a previous child with severe TNMG, AMC, FARIS (FARAD), or recurrent spontaneous miscarriages. For maximal TNMG prevention, it is probably necessary to continue the blockade of antibody transmission until term, but the benefits need to be weighed against potential risks. Increased risks for neonatal infections and fetal hypoalbuminemia represent a concern with FcRn blocker use during pregnancy. Severe hypogammaglobulinemia at birth could be prevented by not administering FcRn blockers during the last month leading up to term, eventually accompanied by IVIG administration, to secure a sufficient dose of non-pathogenic, protective IgG to the child [56]. FcRn therapy, if restricted to gestational weeks 12–33, should still prevent the persistent antenatal damage caused by antibodies to the fAChR (FARIS/FARAD). In this case, TNMG could still occur as this is connected to the adAChR, but the risk for severe TNMG would likely be decreased.

The safety of IVIG monotherapy and TPE during MG pregnancy is documented [98,99]. IVIG and TPE are indicated before and during pregnancy if a mother with MG has had a previous child with TNMG, AMC, or FARIS/FARAD [19].

## 5. Discussion and Future Directions

In total, 10–20% of infants exposed to maternal MG during pregnancy develop TNMG. Compared to other transplacentally transferred autoimmune disorders, this is a high transmission rate. This high maternal-to-fetal “penetrance” of MG illustrates that AChR and MuSK autoantibodies are causal to MG and TNMG and not merely associated biomarkers [43]. TNMG can be a key for understanding other maternal-to-fetal-transferred IgG-mediated diseases, such as systemic lupus erythematosus, Grave’s disease, pemphigus, and immune thrombocytopenic purpura [43].

It is unclear whether it is the absolute antibody dose to the child or certain MG antibody subtypes exceeding a threshold that drives TNMG development. Blocking-type AChR antibodies have pathogenic potential. The role of antibodies against the fAChR in the pathogenesis of TNMG is not clear. Fetal factors modulating the risk are important but not fully understood.

The AChR antibody tests in routine clinical use do not distinguish between different AChR antibody subtypes. Such tests are currently only carried out in research laboratories. The disease mechanisms for TNMG are more heterogenous than for FARAD. This has hampered the development of predictive TNMG markers.

A biomarker that could predict TNMG prenatally or even preconceptionally would be useful. This could guide maternal MG treatment. The consequences of any findings indicating TNMG, such as reduced fetal movement on prenatal ultrasound scans, should be the initiation of preventive therapy in the mother to reduce the IgG antibody load to the child, i.e., IVIG or TPE, or in the future, perhaps FcRn blockers [19]. Mothers with a previous child with TNMG, AMC, FARIS/FARAD, or unstable MG during pregnancy should be followed with serial ultrasound scans as this is the best prenatal predictive tool for TNMG available.

All children with maternal MG exposure need to be observed for at least three days in the hospital. We recommend that an even longer observation period should be practiced in the presence of known risk factors, such as a previously affected sibling or pyridostigmine treatment during delivery. A predictive model to identify asymptomatic newborns that develop TNMG with delay is not available. Such a model would be welcome as it could guide some children to be safely discharged early. Unnecessary interventions would be avoided in healthy newborns, and resources could be targeted to newborns at risk.

It is unclear if delayed TNMG onset can be caused by the lactational transfer of MG antibodies. Very few studies have measured maternal and neonatal antibodies in relation to TNMG development, and even fewer have measured this in both mature milk and colostrum. TNMG could be a model disease for clarifying this matter, as well as lactational IgG transfer in general.

## 6. Conclusions

TNMG is a self-limiting neonatal disease affecting the children of women with clinical or subclinical MG. Life-threatening respiratory depression is the most feared manifestation. All children at risk need to be observed in the hospital for at least 3 days. The disease is treatable and, to some degree, preventable. Good disease control, previous thymectomy, immunosuppressive treatment of the mother, and IVIG/TPE during pregnancy can reduce the risk. TNMG is a model disease of maternofetally transmitted antibody-mediated disorders. TNMG can occur without clinical maternal MG, but this is rare. Hypotonia, a weak cry and weak sucking in an otherwise alert child, should raise the suspicion. Antibodies against AChR or MuSK are usually measurable in the cord blood of neonates with maternal MG exposure, regardless of TNMG symptom expression. The antibodies dissipate spontaneously within days or weeks and, at the latest, within 4 months, parallelled by symptom resolution. Mild TNMG can be overlooked but still become a barrier to breastfeeding success. Neostigmine or pyridostigmine can be used to reduce symptoms. Tube feeding, IVIG, and sometimes respiratory support are needed in more severe cases of TNMG.

## Figures and Tables

**Table 1:** TNMG symptoms by severity grade and our suggested treatment ladder.

Severity Grade	Symptoms	Relevant Treatment Options
Very mild	Fluctuating mild hypotonia. Oral feeding is possible.	Close observation and breastfeeding support.
Mild	Persistent or intermittent hypotonia with feeding difficulties.	Consider low-dose acetylcholine-esterase inhibitor before feedings based on the result of the pharmacological challenge test.
Moderate	Oral feeding is inadequate but there is no respiratory distress.	Nasogastric tube feedings. Acetylcholine-esterase inhibitor. Consider IVIG.
Severe	Respiratory distress.	Acetylcholine-esterase inhibitor regularly. Respiratory support. IVIG and/or TPE.

Abbreviations: TNMG, transient neonatal myasthenia gravis; IVIG, intravenous immunoglobulin; and TPE, therapeutic plasma exchange.

## Data Availability

Not applicable.

## Appendix A

**Table A1:** Table of search strategy and results.

Date	Database	Keywords/Search Strategy	Titles Screened	Articles Considered Relevant/Included
5 June 2023	Cochrane Library	(myasthenia gravis): ti,ab,kw (Word variations searched)	10	2
13 September 2023 + additional auto-alert until 30 November 2023 (results not shown in table)	Embase <1974 to 12 September 2023> Ovid MEDLINE(R) ALL <1946 to 12 September 2023>	myast*.ab,ti. 46,069 offspring*.ab,ti. 196,598 child*.ab,ti. 3,727,412 infan*.ab,ti. 1,085,680 neonat*.ab,ti. 718,460 antenat*.ab,ti. 112,493 prenat*.ab,ti. 269,149 fetal*.ab,ti. 617,511 fetus*.ab,ti. 265,016 foetal*.ab,ti. 46,572 foetus*.ab,ti. 25,696 newborn*.ab,ti. 401,758 progeny*.ab,ti. 77,214 descendant*.ab,ti. 14,440 1 and 2 51 1 and 3 2176 1 and 4 776 1 and 5 942 1 and 6 35 1 and 7 65 1 and 8 506 1 and 9 126 1 and 10 53 1 and 11 17 1 and 12 290 1 and 13 13 1 and 14 0 15 or 16 or 17 or 18 or 19 or 20 or 21 or 22 or 23 or 24 or 25 or 26 or 27 3665 exp congenital myasthenic syndrome/ 2181 28 not 29 3080 “neonatal Fc receptor”.mp. [mp=ti, ab, hw, tn, ot, dm, mf, dv, kf, fx, dq, bt, nm, ox, px, rx, ui, sy, ux, mx] 2296 30 not 31 2915 limit 32 to (english or finnish or danish or icelandic or norwegian or swedish) 2365 limit 33 to full text 440 remove duplicates from 34 290	290	150 (122 English full texts retrieved)
9 June 2023	HELDA—Digital Repository of the University of Helsinki (https://helda.helsinki.fi/. Accessed on 9 June 2023)	(myast*) and (neonat* or infan* or offspring* or fetal* or foetal* or foetus* or fetus* or baby* or babies* or prenat* or antenat* or child* or newborn* or laps* or lasten* or vastasyntyn* or vauv*)	147	2
9 June 2023	https://researchportal.helsinki.fi/. Accessed on 9 June 2023.	(myast*) and (neonat* or infan* or offspring* or fetal* or foetal* or foetus* or fetus* or baby* or babies* or prenat* or antenat* or child* or newborn* or laps* or lasten* or vastasyntyn* or vauv*)	29	0
9 June 2023	Aikakausikirja Duodecim duodecimlehti.fi	(myast*) and (neonat* or infan* or offspring* or fetal* or foetal* or foetus* or fetus* or baby* or babies* or prenat* or antenat* or child* or newborn* or laps* or lasten* or vastasyntyn* or vauv*)	132	2
14 June 2023	MEDIC-tietokanta https://www.fimnet.fi/cgi-cug/medic.pl. Accessed on 14 June 2023.	(myast*) and (neonat* or infan* or offspring* or fetal* or foetal* or foetus* or fetus* or baby* or babies* or prenat* or antenat* or child* or newborn* or laps* or lasten* or vastasyntyn* or vauv* or barn* nyfö* spädbarn* foster* fostre* avkomma*)	8	0
14 June 2023	Suomen Lääkärilehti kokotekstitietokantana vuodesta 1992	(myast*) and (neonat* or infan* or offspring* or fetal* or foetal* or foetus* or fetus* or baby* or babies* or prenat* or antenat* or child* or newborn* or laps* or lasten* or vastasyntyn* or vauv* or barn* or nyfö* or spädbarn* or foster* or fostre* avkomma*)	22	2
14 June 2023	Duodecim-lehti kokotekstitietokantana vuodesta 1992–2007	(myast*) and (neonat* or infan* or offspring* or fetal* or foetal* or foetus* or fetus* or baby* or babies* or prenat* or antenat* or child* or newborn* or laps* or lasten* or vastasyntyn* or vauv* or barn* or nyfö* or spädbarn* or foster* or fostre* or avkomma*)	29	2
14 June 2023	SveMed+ 1977–2019	myast* 35 barn* OR nyfö* OR spädbarn* OR foster* OR fostre* OR avkomma* OR nyfø* OR spedbarn* OR baby* 17,348 #1 AND #2 2	2	1

**Table A2:** Table of TNMG severity grading systems by different authors.

Morel et al., 1988 [42]		Gveric-Ahmetasevic et al., 2008 [23]		Gardnerova et al., 1997 [67]	
Benign	Poor sucking, hypotonia	Grade 1	Weak crying and hypomimia.	Grade 1	Slight hypotonia
		Grade 2	Poor sucking and swallowing, and muscular weakness	Grade 2	Severe hypotonia
				Grade 3	Suction problems
Severe	+ respiratory distress needing mechanical ventilation	Grade 3	Generalized hypotonia with breathing difficulties and respiratory insufficiency	Grade 4	Assisted ventilation
